# Knowledge, Barriers, and Future Directions of Vestibular Rehabilitation Practice in Neurorehabilitation: An Italian Survey

**DOI:** 10.3390/healthcare13010022

**Published:** 2024-12-25

**Authors:** Nicola Ferri, Giovanni Morone, Jacopo Piermaria, Leonardo Manzari, Andrea Turolla, Antonio De Tanti, Irene Ciancarelli, Paolo Pillastrini, Marco Tramontano

**Affiliations:** 1 Department of Biomedical and Neuromotor Sciences, University of Bologna, 40138 Bologna, Italy; nicola.ferri11@unibo.it (N.F.); andrea.turolla@unibo.it (A.T.); paolo.pillastrini@unibo.it (P.P.); marco.tramontano@unibo.it (M.T.); 2 Department of Life, Health and Environmental Sciences, University of L’Aquila, 67100 L’Aquila, Italy; irene.ciancarelli@univaq.it; 3 San Raffaele Sulmona Institute, 67039 Sulmona, Italy; 4 Santa Lucia Foundation, Scientific Institute for Research and Health Care, 00179 Rome, Italy; jacopo.piermaria@gmail.com; 5 MSA ENT Academy Center, 03043 Cassino, Italy; lmanzari1962@gmail.com; 6 Unit of Occupational Medicine, IRCCS Azienda Ospedaliero-Universitaria di Bologna, 40138 Bologna, Italy; 7 Centro Cardinal Ferrari, 43012 Fontanellato, Italy; antonio.detanti@centrocardinalferrari.it

**Keywords:** vestibular rehabilitation, neurological disorders, survey, evidence-based practice, rehabilitation, balance

## Abstract

**Background/Objectives:** Vestibular rehabilitation, an evidence-based physical therapy approach, plays a crucial role in managing and recovering from gaze and balance disorders, including those of central origin. This study, targeted at the community of Italian healthcare practitioners, is vital in understanding the application of vestibular rehabilitation in neurological disorders and in identifying knowledge gaps, barriers, and future directions. **Methods:** This is a cross-sectional study directed at healthcare professionals involved in neurorehabilitation in Italy. The survey consisted of 29 items grouped in 4 sections, which was estimated to take approximately 10 min to complete. The questions covered socio-demographic information, professional information, clinical practice, and future perspectives on vestibular rehabilitation. **Results:** Out of the 435 respondents, 290 completed the survey. Most of the respondents reported either no (32.87%) or little (42.91%) experience in vestibular rehabilitation. However, most participants (72.98%) recognized the importance of vestibular rehabilitation in treating neurological disorders. The most common condition treated was stroke (46.39%), while balance training (52.69%) and visual input exercises (26.35%) were the two most frequently used strategies. The main barriers to implementing vestibular rehabilitation in clinical practice were equipment cost and insufficient skills. **Conclusions:** Vestibular physical therapy is a promising complementary approach in neurorehabilitation. However, the study reveals a perceived lack of basic training in vestibular assessment and therapy. This suggests that more efforts are needed to bridge this knowledge gap and make necessary equipment more accessible.

## 1. Introduction

Vestibular physical therapy (VPT), otherwise known as vestibular rehabilitation (VR), is an evidence-based physical therapy approach to managing and recovering from gaze and balance stability disorders through specific exercises targeting central compensation [1]. Initially aimed at peripheral vestibular disorders, the literature has provided important evidence on its effectiveness in the functional recovery after unilateral and/or bilateral peripheral vestibular pathologies [2,3]. Recently, new evidence suggested the significant role of VPT in the treatment of disorders also of central origin [4].

For instance, in multiple sclerosis, VPT interventions, such as vestibulo-ocular reflex training, have demonstrated efficacy in reducing dizziness and improving postural stability [5]. Similarly, in Parkinson’s disease, tailored exercises incorporating static and dynamic balance training have been shown to enhance gait, reduce fall risk, and improve quality of life [6].

People recovering from traumatic brain injury (TBI) also benefit significantly from VPT, as it helps mitigate persistent dizziness, motor coordination challenges and activities of daily living through gaze and postural stability exercises [7,8,9]. In pediatric populations, children with cerebral palsy have exhibited marked improvements in motor functions and sensory integration when engaged in vestibular rehabilitation programs that incorporate playful yet targeted tasks to stimulate the vestibular system [10].

The versatility of VPT extends to stroke rehabilitation [11], where it enhances gait symmetry and postural control, particularly in individuals with balance impairments due to central nervous system damage. These advancements underscore VPT’s broad applicability and the growing recognition of its importance as an integral component of neurorehabilitation programs.

Neurological disorders are the leading cause of disability worldwide [12] and a prevalent reason for the demand for rehabilitation services [13], with major economic consequences on healthcare systems [14].

Despite this growing body of evidence in support of VPT, recent surveys inform us of a lack of accuracy and training for the management of vestibular disorders in people after traumatic brain injury [15], limited referral from medical doctors [16], major discrepancies in vestibular clinical practice [17], challenges in vestibular knowledge translation and implementation [18], and underuse of canalith repositioning maneuvers and VR from general practitioners [19].

A recent survey [20] investigated VR practice and research in Europe reporting good knowledge of vestibular disorders treatment, but important heterogeneity in vestibular assessment.

This research aims to map the knowledge and use of VPT in neurological disorders by healthcare professionals working in neurorehabilitation in Italy and to highlight knowledge, barriers, and future perspectives.

## 2. Materials and Methods

### 2.1. Study Design

This cross-sectional study was approved by the Bioethics Committee at XXX and directed to healthcare professionals involved in neurorehabilitation (i.e., physicians, physiotherapists, occupational therapists, developmental therapists), in Italy. The full survey protocol has been published on medRxiv [21]. The questionnaire consists of 4 sections: socio-demographic information, professional information, clinical practice and future perspectives. It included 27 closed and 2 open questions.

Five experts created the draft survey with an iterative feedback process to enhance response time, validity, clarity, and fluency. Six independent respondents who are representatives of our target audience piloted the draft survey, after which the final version (Supplementary File S1) was generated by incorporating the collected feedback. The demographic characteristics of the participants who piloted the questionnaire are reported in Table 1. The estimated time to complete our survey was approximately 10 min.

The survey was developed using Qualtrics XM software–version July 2024 (Qualtrics, Provo, UT, USA, 2020) [22] and has been conducted in adherence with Dillman’s approach [23] to enhance compliance. Qualtrics automatically screened the responders based on their IP addresses to prevent different responses from the same person. Before the survey began, informed consent was acquired, and no personally identifiable information was gathered. National professional associations sent out emails containing the survey link. No payment or incentive was given to the respondents.

We estimated a sample size of 275 respondents based on our calculations.

### 2.2. Statistical Analyses

Descriptive analyses are reported for continuous variables as mean and standard deviation (SD), or median and interquartile range (IQR) according to distribution properties, while categorical variables are reported as counts and percentages. Data normality was tested by the Shapiro–Wilk test. The two open-ended questions were manually coded through thematic analyses after key themes were identified; the results were reported as word clouds, with word size representing the frequency of the tag applied to each answer of the two questions.

Statistical analyses were performed using STATA SE 18 (StataCorp. 2023, College Station, TX, USA) [24].

We conducted this study following the methodological recommendations available, [23,25] and we reported it in accordance with the Checklist for Reporting Of Survey Studies (CROSS) [26] (Supplementary File S2).

## 3. Results

### 3.1. General Results

A total of 20,000 emails were sent through the leading professional and scientific Italian organizations representing the population of interest. Finally, 435 people opened the survey link, 18 denied consent to the survey, and 127 were blank surveys. As a result, 290 responses were included in the analyses. Sample characteristics are reported in Table 2.

**Figure 1:**
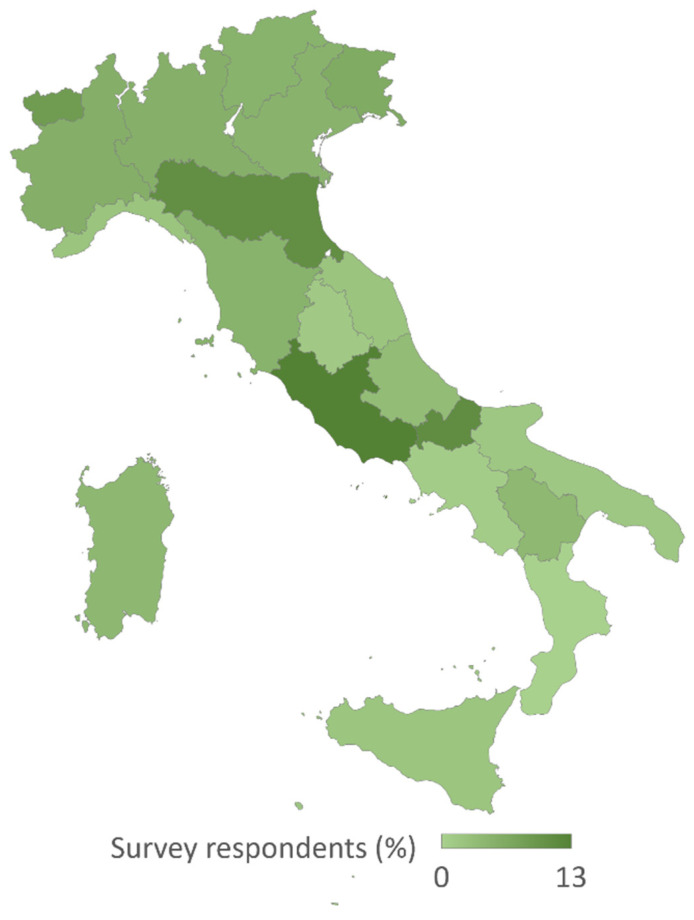
Respondent rate adjusted for the regional population.

The respondents’ geographic locations cover the entire Italian peninsula (Figure 1). The median response time was 8 min (IQR 8).

### 3.2. Education and Expertise

The respondents had a median work experience of 10 years (IQR 15) and a median academic education of 5 years (IQR 5).

Overall, 70 (24.22%) respondents reported full expertise in vestibular rehabilitation, 124 (42.91%) reported little expertise, and 95 (32.87%) no expertise. In contrast, 72.98% of the respondents were aware of the role of vestibular rehabilitation for people with neurological disorders. Of the 290 who completed the survey, some items were missing, particularly in the domain of clinical practice. Analysis by subgroup revealed that the completion rate was lower in those who reported no expertise. Indeed, item 29 “There is a need for more vestibular rehabilitation education during the basic academic course” had a completion rate of 36% for no-expertise, 58% for little-expertise, and 78% for full-expertise respondents.

**Figure 2:**
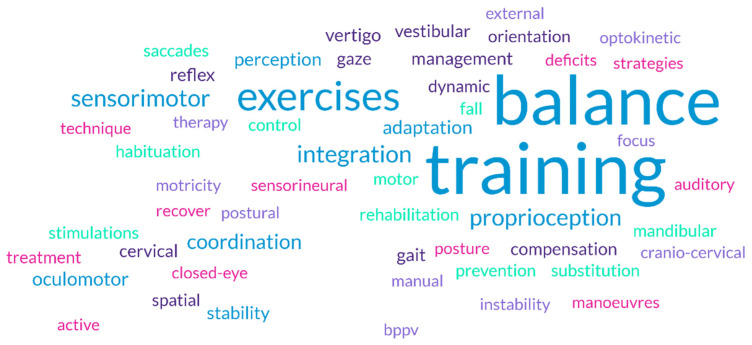
Vestibular rehabilitation definition. Word cloud.

Figure 2 illustrates the thematic analysis of item 13, “According to your knowledge, what is meant by vestibular rehabilitation?”; in the Supplementary File S3 the frequency analyses are reported.

### 3.3. Clinical Practice

Physiatrists were the most frequent prescriber of rehabilitation in the 41.84% of respondents, followed by neurologists (26.95%), otolaryngologists (21.99%), general practitioners (6.38%), and internists (2.84%). Almost half of the respondents (46.33%) do not use or consider instrumental assessments such as electronystagmography, videonystagmoscopy, videonystagmography, video head impulse test, vestibular evoked myogenic potentials, Frenzel goggles, dynamic plates, or rotator chairs, and 16.38% of respondents use dynamic plates only.

Stroke was the most frequent CNS disorder referred to vestibular rehabilitation in 46.39% of respondents, followed by Parkinson’s disease (16.87%), multiple sclerosis (15.06%), brain injury (6.02%), cerebral palsy (3.01%), and mild cognitive impairment (0.60%).

The most frequent strategies used by respondents are balance training (52.69%) and visual input exercises (26.35%); in the case of aVOR deficit, the choice of stimulating the deficit reflex, compensatory saccades, and the optokinetic reflex is evenly distributed, with approximately 19% of respondents always stimulating them, 44–47% stimulating them sometimes, and 33–36% never using these types of strategies.

Overall, 79.17% of respondents stimulate gaze stability with both static and dynamic strategies (i.e., during walking training), and 5.95% stimulate it exclusively in a sitting position. Walking on the treadmill with eyes closed to promote greater stability during the stride is proposed by 65.88% of the respondents.

Most respondents treat patients with VPT once or twice a week (78.57%), for a duration between 15 and 30 min (55.13%).

**Figure 3:**
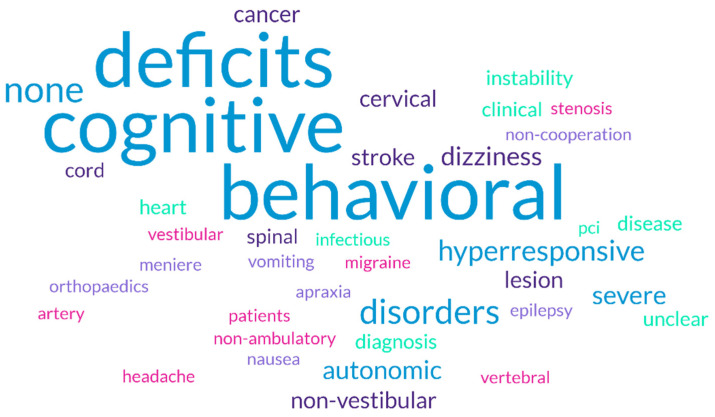
Limitations to vestibular rehabilitation. Word cloud.

Figure 3 reported the reasons why respondents avoid or limit the use of VPT in their practice (item 27), resulting in a clear prevalence of cognitive and behavioral aspects. The complete reporting of the frequency analyses can be found in Supplementary File S3.

### 3.4. Current Perception and Future Perspectives

**Figure 4:**
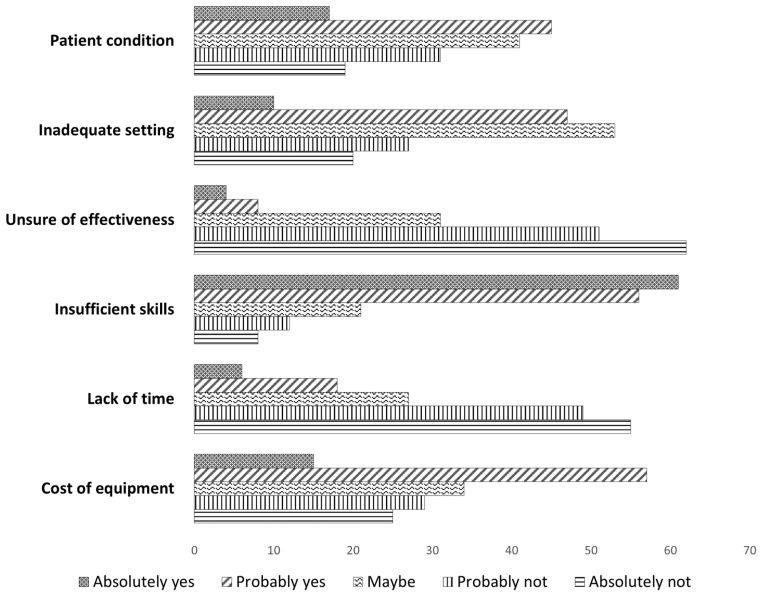
Are these barriers to the use of VPT?

Respondents perceived the cost of equipment and insufficient skills as the major barriers to use VPT during their clinical practice (Figure 4).

Overall, 73.01% of respondents completely agreed that more vestibular rehabilitation education should be provided during the basic academic course. Furthermore, there is a widespread opinion that specific guidelines for vestibular rehabilitation in neurological disorders are needed, with 62.20% of respondents completely agreeing and 31.10% fairly agreeing.

## 4. Discussion

The impact of neurological disorders on the quality of life of people, their families, and healthcare systems’ budgets is well known [12,14]. On the other hand, the scientific literature provides us with a growing body of evidence on treatment effectiveness, which is increasing exponentially from year to year [27]. This results in the risk of latency in knowledge transfer to clinical practice, which is also related to the difficulty of managing and selecting the best evidence when there is so much productivity on the same topic [28].

Numerous surveys have highlighted challenges regarding vestibular assessment and rehabilitation [15,16,17,18,19,20]. Indeed, VR is an approach that originated in the late 1940s with Cawthorne’s [29] and Cooksey’s [30] habituation exercises for vestibular disorders, but it has been constantly enriched over the decades by radically changing its approach and application with new knowledge, equipment, and clinical advances [31].

Indeed, our survey highlighted a perceived need for updating academic training both for PM&R physicians, Neurologists and Otologists, Physiotherapists, Occupational therapists and Developmental therapists. This consideration is also supported by the fact that VR, which was developed for very selective vestibular disorders, now has applications in a wide spectrum of neurological and non-neurological disorders [31]. This lack of specific skills is not only related to the responses to the specific question, but is also supported by the low completion rate, which could be related to less skilled respondents.

The patient’s condition is indicated among the possible barriers in the use of VR. It is important to emphasize this aspect as not all patients can benefit from VR. In the context of neurorehabilitation, a specific range of motor and cognitive functions can be included in treatments. These differences must be adapted for the pathology that causes that alteration of function [4,5,6,7,8] (i.e., Parkinson Disease, Stroke, Multiple Sclerosis). It will be important in clinical indications and future guidelines to correctly indicate the inclusion criteria in terms of clinical and functional conditions (motor and cognitive) for treatments and to provide specific indications of VR adaptation matching subjects’ functionality. Studies have emphasized the role of specific exercises, including gaze and postural stability training, in improving outcomes for these conditions. In particular, current research reported sensory input rebalance exercises (e.g., eyes open vs. eyes closed) and aVOR training with different types of surfaces and stances for people with multiple sclerosis, traumatic brain injury, Parkinson’s disease, and cerebral palsy [8,10,32,33,34]. However, despite these advancements, gaps persist in translating this knowledge into clinical practice, as revealed in our survey. Healthcare practitioners often face barriers such as a lack of training and resources, limiting their ability to implement these evidence-based strategies.

The inadequate setting is also a possible barrier. It should be emphasized that VR is indicated for patients both in the subacute and chronic stabilized phases and does not require a setting that is particularly different from that of normal functional rehabilitation. People with neurological conditions could benefit from a sensory rebalance approach by tailoring the exercise to the patient’s abilities, training on different surfaces and stances, with eyes closed, or with visual targets, and integrating the information from the various systems with, for example, active head turning to engage the vestibular system as well, possibly adding cognitive engagement with double tasks and goal-oriented requests. This is a very low-cost approach, without the need for settings other than the usual rehabilitation context. Thus, the perceived barrier probably reflects the knowledge-to-action gap in the practice and setting of VR exercises.

Moreover, there is strong agreement that uncertainty about effectiveness and the lack of time are not barriers to the spread of VR. These responses seem to imply the awareness that VR is an unmet need for standard care.

Finally, insufficient skills and equipment costs are indicated as possible barriers to the diffusion of VR. These two aspects may reflect the need to increase knowledge about VR in general, especially what is needed for service delivery, and there is a need to increase the curricula of rehabilitation health professionals at all levels, i.e., academics, scientific societies, and professional associations.

It should be considered that VR and minimal instrumental assessment are low-cost strategies and require a simple setting. However, there might still be a need to create geographically distributed centers where patients could take advantage of more complex, expensive, but accurate assessment tools. One possible solution is the creation of Hub and Spoke centers [35], where both the instrumentation and the presence of qualified specialists could allow the best patient care for the most complex cases of vestibular dysfunctions.

It is worth highlighting that this is the first study with a large and representative sample to map healthcare professionals’ knowledge and clinical practice in VPT for persons with central nervous system disorders in Italy.

Although representative of the various professions, the sample is undersized compared to the estimated number of professionals on the ground. Currently, it is still difficult to understand whether this is a recruitment problem or if there is a lack of specific expertise in the community. To reach out all the potential clinicians, the survey was sent to a large population of eligible professionals (i.e., global national registries) enhancing the chance of reaching clinicians involved in neurorehabilitation, but not registered in the special sections of associations, thus justifying the low response rate. A second limitation is the presence of 127 blank surveys, which could be related to a lack of time, interest, or expertise. Lastly, the regional distribution of respondents indicates a general coverage of Italy, albeit with some heterogeneity. Although we cannot exclude other reasons, this finding could be related to Italy’s healthcare system, which is based on regional autonomy, thus with different resources, number of hospital beds, and healthcare facilities in each territory.

## 5. Conclusions

This study provides a comprehensive overview of vestibular rehabilitation practice in neurorehabilitation across Italy, highlighting significant knowledge gaps, barriers, and future directions. Despite growing evidence supporting vestibular rehabilitation as an effective approach to managing neurological disorders, our findings reveal a perceived lack of basic training in vestibular physical therapy assessment and rehabilitation among healthcare professionals. Future efforts should be made regarding knowledge translation and accessibility of specific equipment.

## Figures and Tables

**Table 1:** Piloting respondents (n = 6).

	Piloting Respondents
Sex (Female, %)	3 (50%)
Age (mean, SD)	41.8 (10.7)
Professional role * (n, %)	
Clinician	5 (83%)
Researcher	2 (33%)
Academic education (years, SD)	6.2 (3.1)

* Total > 6 because respondents may be clinicians and researchers.

**Table 2:** Sample characteristics.

Characteristic	Sample
Female (%)	60.63%
Age (median, IQR)	39.5 (19)
Job title (%)	
Physical therapist	77.35%
Physician	16.03%
Occupational therapist	4.53%
Developmental therapist	2.09%
Professional role (%)	
Clinician only	74.74%
Clinician and researcher	23.86%
Researcher only	1.40%
Employment type (%)	
Independent	39.45%
Public	36.68%
Private	23.88%

## Data Availability

Data are contained within the article and Supplementary Materials.

## Supplementary Materials

The following supporting information can be downloaded at: https://www.mdpi.com/article/10.3390/healthcare13010022/s1, Supplementary File S1. Survey. Supplementary File S2. CROSS checklist. Supplementary File S3. Frequency Tables.
