# Supplementary material for: Knowledge, Barriers, and Future Directions of Vestibular Rehabilitation Practice in Neurorehabilitation: An Italian Survey
Source: Healthcare (Basel). 2024 Dec 25;13(1):22. doi: 10.3390/healthcare13010022 (PMC11719692; doi:10.3390/healthcare13010022)
Supplement: Supplementary file 1 [file healthcare-13-00022-s001.zip › Supplementary File 1.pdf]

Supplementary File 1

Riabilitazione vestibolare in neuroriabilitazione

Inizio blocco: Consenso informato

Gentile Collega,
Stiamo conducendo uno studio di ricerca presso il Dipartimento di Scienze Biomediche e Neuromotorie dell'Università di Bologna, volto a valutare il livello di conoscenza e di adozione della riabilitazione vestibolare nel trattamento di persone affette da patologie neurologiche. Siamo convinti che la sua esperienza e competenza possano apportare un contributo di valore significativo al nostro studio, pertanto le saremmo grati se potesse prendere parte a questa ricerca partecipando ad un breve questionario, che richiederà solo pochi minuti del suo tempo. Il suo coinvolgimento sarà di grande importanza per il successo e la validità dei risultati ottenuti. Restiamo a disposizione per fornire ulteriori informazioni e chiarimenti in merito al progetto. La ringraziamo fin d'ora per la sua attenzione e per la considerazione della nostra richiesta. Cordiali saluti

Q1 Informativa privacy

- o Acconsento
- o Non acconsento

Salta a: Fine sondaggio Se Consenso informato = Non acconsento

Fine blocco: Consenso informato

Inizio blocco: Informazioni socio-demografiche

Q2 Età:
Anni ____

Q3 Sesso:

- Maschio
- Femmina

Q4 Genere:

- Uomo
- Donna
- Genere non-binario / Terzo genere
- Preferisco non dirlo

Q5 In quale provincia svolgi la tua attività professionale?

▼

Fine blocco: Informazioni socio-demografiche

Inizio blocco: Informazioni professionali

Q6 Il mio rapporto di lavoro prevalente è:

- Dipendente pubblico
- Dipendente privato
- Libero professionista

Q7 Il mio profilo professionale è:

- Medico
- Fisioterapista
- Terapista occupazionale
- TNPEE

Q8 Mi identifico come un:

- Clinico
- Ricercatore
- Entrambi

Q9 Lavoro in neuroriabilitazione da:

	0	5	10	15	20	25	30	35	40	45	50
(n° anni) ()											

Q10 La mia formazione accademica ha avuto una durata complessiva di:

	0	2	3	5	6	8	9	11	12	14	15
(n° anni) ()											

Q11 Hai esperienza di riabilitazione vestibolare?

- No
- Poco
- Sì

Q12 Sei a conoscenza del ruolo della riabilitazione vestibolare per i pazienti con disordini neurologici?

- No
- Sì

Q13 Secondo le tue conoscenze, cosa si intende per riabilitazione vestibolare?

____

Q14 La riabilitazione vestibolare era parte del mio core curriculum.

- Falso
- Vero

Q15 La riabilitazione vestibolare è parte della mia pratica clinica attuale.

- Falso
- Vero

Q16 Nella mia pratica clinica mi occupo di:

- ☐ Rieducazione dell'equilibrio
- ☐ Rieducazione del cammino
- ☐ Prevenzione delle cadute

Q17 Il mio setting clinico prevalente è costituito da:

- o Day Hospital
- o Ambulatoriale
- o Territoriale
- o Studio privato
- o Laboratorio di ricerca
- o Domiciliare
- o RSA

Fine blocco: Informazioni professionali

Inizio blocco: Pratica clinica

Q18 La maggior parte delle prescrizioni proviene da (ordina le risposte dalla più frequente alla meno frequente):

______ ORL
______ Neurologo
______ Fisiatra
______ MMG
______ Internisti

Q19 Nella mia pratica clinica eseguo e/o considero le valutazioni fatte da altri con i seguenti strumenti:

- ▢ ENG (elettronistagmografia)
- ▢ VNS (videonistagmoscopia)
- ▢ VNG (videonistagmografia)
- ▢ vHIT (video Head Impulse Test)
- ▢ VEMPS (potenziali evocati vestibolari)
- ▢ Occhiali di Frenzel
- ▢ Pedane di equilibrio dinamico
- ▢ Sedia rotatoria
- ▢ Nessuno delle precedenti

Q20 Mi occupo di riabilitazione vestibolare soprattutto nei pazienti con (ordina le risposte dalla più frequente alla meno frequente):

- ▢ ______ Ictus
- ▢ ______ Sclerosi multipla
- ▢ ______ Malattia di Parkinson
- ▢ ______ Trauma cranico
- ▢ ______ Paralisi cerebrale infantile
- ▢ ______ Mild cognitive impairment
- ▢ ______ Altro

Q21 Le strategie di riabilitazione vestibolare che utilizzo maggiormente sono (ordina le risposte dalla più frequente alla meno frequente):

______ Esercizi a input visivo (es., esercizi di Herdman)
______ Training dell'equilibrio
______ Sedia rotatoria
______ Accelerazioni lineari
______ Stimolazioni otticocinetiche
______ Realtà virtuale
______ Manovre di Brandt-Daroff
______ Training del cammino
______ Rilassamento (es., respirazione, yoga, etc.)
______ Training percettivo

Q22 In caso di deficit del VOR, sono solito stimolare:

	Mai	A volte	Sempre
Il riflesso deficitario	o	o	o
Le saccadi compensatorie	o	o	o
Il riflesso otticocinetico	o	o	o

Q23 Nella mia pratica clinica, la stabilità dello sguardo la stimolo:

- o solo da seduto
- o da seduto e da in piedi
- o sia in statica che in dinamica, stimolandola anche durante il cammino richiedendo al paziente la ricerca di target nello spazio

Q24 Nella tua pratica clinica inserisci esercizi ad occhi chiusi sul treadmill per stimolare una maggiore stabilità durante il passo?

- o No
- o Sì

Q25 Con quale frequenza i tuoi pazienti effettuano riabilitazione vestibolare?

- o 1 v. / settimana
- o 2 vv. / settimana
- o 3 vv. / settimana
- o 4 vv. / settimana
- o 5+ vv. / settimana

Q26 Nella tua pratica clinica, con quale durata è effettuata la riabilitazione vestibolare all'interno di una singola seduta?

- o < 15'
- o tra 15' e 30'
- o > 30'

Q27 Con quali pazienti o disabilità eviteresti o limiteresti l'uso della riabilitazione vestibolare?

____

Fine blocco: Pratica clinica

Inizio blocco: Prospettive future

Q28 Consideri i seguenti aspetti delle barriere per l'utilizzo della riabilitazione vestibolare?

	Assolutamente no	Probabilmente no	Forse	Probabilmente sì	Assolutamente sì
Costo strumentazione	o	o	o	o	o
Mancanza di tempo	o	o	o	o	o
Competenze insufficienti	o	o	o	o	o
Non sicuro dell'efficacia	o	o	o	o	o
Setting non adeguato	o	o	o	o	o
Condizioni del paziente	o	o	o	o	o

Q29 C'è la necessità di una maggiore educazione alla riabilitazione vestibolare durante il percorso accademico di base.

- o Completamente in disaccordo
- o Abbastanza in disaccordo
- o Né d'accordo né in disaccordo
- o Abbastanza d'accordo
- o Completamente d'accordo

Q30 C'è la necessità di lineeguida per la riabilitazione vestibolare nei disturbi neurologici.

- o Completamente in disaccordo
- o Abbastanza in disaccordo
- o Né d'accordo né in disaccordo
- o Abbastanza d'accordo
- o Completamente d'accordo

Fine blocco: Prospettive future
