# Supplementary material for: Knowledge, Barriers, and Future Directions of Vestibular Rehabilitation Practice in Neurorehabilitation: An Italian Survey
Source: Healthcare (Basel). 2024 Dec 25;13(1):22. doi: 10.3390/healthcare13010022 (PMC11719692; doi:10.3390/healthcare13010022)
Supplement: Supplementary file 1 [file healthcare-13-00022-s001.zip › Supplementary File 3.pdf]

Supplementary File 3

Item 13. Vestibular rehabilitation definition	Counts	%
Balance training	97	47.1
Sensorimotor integration	15	7.3
Proprioception exercises	14	6.8
Coordination exercises	9	4.4
Oculomotor exercises	6	2.9
Adaptation	6	2.9
Perception	5	2.4
Vertigo management	4	1.9
Gait training	4	1.9
Compensation exercises	3	1.5
Cervical exercises	3	1.5
Gaze stability	3	1.5
Reflex exercises	3	1.5
Saccades rehabilitation	2	1.0
Spatial orientation	2	1.0
Exercises	2	1.0
Fall prevention	2	1.0
Motor control	2	1.0
Habituation	2	1.0
Substitution	2	1.0
Mandibular exercises	2	1.0
External focus	1	0.5
Postural instability	1	0.5
Optokinetic training	1	0.5
Manual therapy	1	0.5
Motricity	1	0.5
Auditory exercises	1	0.5
Cranio-cervical treatment	1	0.5
Dynamic exercises	1	0.5
Vestibular stimulations	1	0.5
Manoeuvres	1	0.5
Sensorineural stimulations	1	0.5
Technique to recover from deficits	1	0.5
Active strategies	1	0.5
Vestibular exercises	1	0.5
Closed-eye exercises	1	0.5
Vestibular reflex training	1	0.5
Dynamic posture stability exercises	1	0.5
BPPV treatment	1	0.5
**Total**	206	100

Item 27. Vestibular Rehabilitation barriers	Counts	%
Cognitive behavioral deficits	22	29.7
None	8	10.8
Hyperresponsive	5	6.8
Severe dizziness	4	5.4
Autonomic disorders	3	4.1
Non-vestibular disorders	3	4.1
Cancer	3	4.1
Stroke	3	4.1
Cervical lesion	3	4.1
Heart disease	2	2.7
Spinal cord	2	2.7
Unclear diagnosis	2	2.7
Clinical instability	2	2.7
Epilepsy	1	1.4
Non-cooperation	1	1.4
Ménière	1	1.4
Apraxia	1	1.4
Vertebral artery stenosis	1	1.4
Nausea or vomiting	1	1.4
Headache	1	1.4
Vestibular migraine	1	1.4
Cerebral palsy	1	1.4
Non-ambulatory patients	1	1.4
Infectious	1	1.4
Orthopaedics	1	1.4
**Total**	74	100
